# Global burden of chronic kidney disease due to diabetes mellitus type 2 attributable to low physical activity and high body mass index from 1990 to 2021

**DOI:** 10.5114/biolsport.2025.150045

**Published:** 2025-05-08

**Authors:** Weifeng Pan, Fangbo Li, Xiangyu Du, Mingyan Ye, Songtao Wang, Kexin Shi

**Affiliations:** 1 School of Physical Education and Sports Science, South China Normal University, Guangzhou, China; 2 Department of Physical Education, Northeastern University, Shenyang, China

**Keywords:** Global disease, Diabetic kidney disease, Sedentary behavior, Physical inactivity, Obesity

## Abstract

The present study aimed to assess the global chronic kidney disease due to diabetes mellitus type 2 (CKD-T2DM) attributable to low physical activity (PA) and high body-mass index (BMI). Data on CKD-T2DM deaths, disability-adjusted life years (DALYs), and risk factor exposure were obtained from the Global Burden of Disease Study 2021. The jointpoint model was used to detect the temporal patterns throughout the past 32 years, and the age-period-cohort analyses were preformed to clarify the trends in epidemiological shift. The global age-standardized rate (ASR) of DALYs for CKD-T2DM linked to low PA were 8.19 (95% UI, 3.21 to 13.60) per 100,000 people. No significant percent changes were observed since 1990. Globally, high BMI accounted for 50.14 (95% UI, 22.56 to 79.15) ASR of DALYs with a marked increase of 62.66 %(95 % UI, 44.24 to 78.73) from 1990. Both low PA and high BMI-attributable CKD-T2DM showed a surging trend in population aged less than 40 or greater than 75. There were consistent nonlinear trajectories between socio-demographic index (SDI) and CKD-T2DM attributable to low PA and high BMI, which reached a peak at SDI = 0.6 before decreasing with a higher SDI value. Globally, there are rising trends in CKD-T2DM burden, low PA exposure, and high BMI exposure until 2050. There are secular improvements in CKD-T2DM attributable to low PA, while it remains a severe challenge in that of high BMI. Considerable temporal and regional variation highlight the urgency of structural, context-specific strategies targeted on modifiable factors to tackle the CKD-T2DM burden.

## INTRODUCTION

Chronic kidney disease due to diabetes mellitus type 2 (CKD-T2DM) remains a primary severe diabetic complication, markedly affecting public health worldwide [1]. Recent studies reported that CKD-T2DM was responsible for 11.3 million (95% UI, 9.71 to 13.1) disability-adjusted life years (DALYs) and 25.4% (95% UI, 22.4 to 28.7) of total overall chronic kidney disease DALYs in 2021 [2]. The global CKD-T2DM burden has been ascending over the past three decades, and economic development, paired with population growth, accounts for the most significant increment [1]. Previous studies have demonstrated that regular physical activity is favorable to maintaining overall health by reducing disease risk and premature mortality [3, 4]. Meanwhile, strong evidence has revealed that management of overweight and obesity can prevent noncommunicable diseases such as cardiovascular disease and type 2 diabetes [5–8]. Several studies suggested that there are dose-response relationships between CKD-T2DM and PA and BMI [9–11]. For example, a recent study from a prospective cohort reported that the most physically active individuals had a lower risk of CKD-T2DM compared with the least inactive participants, with a corresponding hazard ratio of 0.75. Another cohort study revealed that a healthy lifestyle, including recommended waist circumference for patients with T2DM, could curb the incidence of diabetic kidney disease with a hazard ratio of (0.43) [9]. Although the benefits of regular PA and weight management on major health outcomes are well documented, the understanding of the association between global CKD-T2DM and these two lifestyle factors remains unclear. On the other hand, recent evidences show that even 27.5% of adults worldwide fail to meet the minimum standard of PA recommended by mainstream public health guidelines [12], while there has been a surging trend in global overweight and obesity prevalence throughout the past three decades [13].

The Global Burden of Disease (GBD) study has made substantial efforts in evaluating longitudinal variation of more than 459 health outcomes and risk factors across 204 countries and territories, which allows for the determination of temporal and regional patterns of global CKD-T2DM burden [2]. Nonetheless, an up-to-date picture of the CKD-T2DM burden attributable to these two lifestyle factors worldwide remains unclear. There is an urgent need to quantify the CKD-T2DM burden attributable to low PA and high BMI using the latest evidence based on a large-scale population. This would help to assess the effects of previous efforts and guide targeted structural interventions for curbing upward trends in global CKD-T2DM. Thus, the present study aims to provide insights into an updated landscape of CKD-T2DM burden attributed to high BMI and low PA worldwide between 1990 and 2021 by sex, age, and development status of regions based on GBD 2021.

## MATERIALS AND METHODS

### Diabetic kidney disease data

Briefly, CKD-T2DM is defined as T2DM-attributable CKD, lasting for more than 3 months, paired with a urinary albumin/creatinine ratio no less than 30 mg · g and/or glomerular filtration rate less than 60 mL · min · 1.73 m^2^ (ICD-10 code E 11.2–E11.29) [14]. The methodologies used to obtain overall CKD-T2DM data were well described in GBD 2021 [2]. The estimation process of GBD aimed at generating a robust and systemic evaluation of health outcomes and risk factors through gathering data in a comprehensive range. The modeling tool DisMod-MR 2.1, based on the Bayesian meta-regression method, was used to simultaneously generate estimates of CKD-T2DM deaths and disability-adjusted life years (DALYs) paired with corresponding 95% uncertainty intervals, which utilized high-quality data from various sources integrating prevalence, incidence, and mortality to obtain a valid and accurate evaluation of CKD-T2DM worldwide [15].

### Definitions

In GBD 2021, a certain low PA exposure level that minimizes all-cause deaths was defined the theoretical minimum-risk exposure levels (TMRELs), which was based on epidemiological and clinical evidence from relative risks by PA level and the lowest PA level from cohort studies [16]. This PA threshold was finally defined as 3000–4500 metabolic equivalent minutes per week which is the minimum number of deaths that could be observed across health outcomes [16]. The TMRELs for high BMI in adults was calculated based on the level of BMI that was associated with the lowest risk of all-cause mortality by updated dose-response risk curves for BMI with the burden of proof meta-analytic approach and dropped one risk-outcome pair [16]. Thus, the high BMI exposure for adults (ages 20 and older) was defined as BMI greater than 20–23 kg · m^2^ [16].

The socio-demographic index (SDI) is a systemic indicator that quantitatively assesses the overall status of socio-demographic development for each area through the utilization of metrics such as the fertility rate among people aged less than 25, the educational attainment among population aged greater than 15 and average income per capita [15]. In brief, SDI ranges from 0 to 1, representing the worst or best social-economic status for each region, respectively. Generally, 204 countries and territories were classified into the following five SDI regions: low SDI (< 0.46), low-middle SDI (0.46 to 0.61), middle SDI (0.61 to 0.71), high-middle SDI (0.71 to .0.81), and high SDI (> 0.81) [15].

### Statistical analysis

Deaths and disability-adjusted life years (DALYs) attributable to PA and high BMI were obtained from GBD 2021 [2]. The DALYs were used to quantify the health loss by summing years lived with disability and years of life lost due to disease. For example, one DALYs means one-year life loss equivalent to full health [2]. The population attributable fractions (PAF) were utilized to estimate the proportion of deaths and DALYs contributed to low PA and high BMI, which means a corresponding proportion that could be decreased if risk factors were fitted to theoretical minimum-risk exposure level [16]. To ensure that the CKD-T2DM burden attributable to low PA and high BMI was comparable across locations and periods, necessary age standardization based on global age structure was performed [17].

For illuminating the age, period, and birth cohort patterns especially the variation of CKD-T2DM burden rate for each age group, the age-period-cohort model including 5-year age groups aligned with 5-year period intervals were used [18]. The present study defined six 5-year temporal intervals spanning from 1990 to 2019 and corresponding 20 birth cohorts from 1895 to 1990. This facilitated a clear investigation of CKD-T2DM trend attributable to low PA and high BMI in age, period, and cohort effects perspective [18]. In addition, the joinpoint model was used to detect a further variation of temporal trends in CKD-T2DM burden contributed to these two factors [19]. Both average annual percent change (AAPC) from 1990 to 2021 and segmental AAPC during specific period across SDI regions were estimated to provide insights into a comprehensive landscape of lifestyle-related CKD-T2DM burden [19].

This study also assessed the association between SDI and age-standardized CKD-T2DM burden attributable to low PA and high BMI using the LOESS regression method [20]. To predict the trend in DALYs rate for CKD-T2DM paired with corresponding low PA and high BMI exposure between 2021 and 2050, we obtained future forecast results using GBD Foresight Visualization (https://vizhub.healthdata.org/gbd-foresight) [21].

All analyses and visualizations were performed in R software (version 4.3.2). Two-sided statistical tests were conducted, and 95% uncertainty intervals excluding zero were considered statistically significant.

### Ethics

No identifiable data were utilized in GBD 2021, therefore, a waiver of informed consent was evaluated and approved by the University of Washington Institutional Review Board [15].

## RESULTS

### Overall impact of low physical activity and high body-mass index

In 2021, the global age-standardized rate (ASR) of deaths and DALYs for CKD-T2DM potentially attributable to low PA were 0.38 (95% UI, 0.15 to 0.63) and 8.19 (95% UI, 3.21 to 13.60) per 100,000 people, respectively. There was no significant percentage change in both the global ASR of deaths and DALYs for low-PA-related CKD-T2DM from 1990 to 2021. Low PA potentially contributed to 6.55% (95% UI, 2.71 to 10.72) of deaths and 6.25% (95% UI, 2.51 to 10.22) DALYs for CKD-T2DM in 2021, corresponding a decrease of -18.30% (95% UI, -24.44 to -11.47%) and -16.29 (95% UI, -22.34% to -9.36) since 1990, respectively. In contrast, high BMI potentially accounted for 2.07 (95 % UI, 0.91 to 3.47) ASR of deaths and 50.14 (95% UI, 22.56 to 79.15) ASR of DALYs in 2021, and corresponding a marked increase of 79.26% (95 % UI, 55.59 to 98.31) and 62.66 %(95 % UI, 44.24 to 78.73) from 1990, respectively. In 2021, 36.18% (95 % UI, 15.97 to 57.93) deaths and 38.24% (95 % UI, 17.54 to 58.90) DALYs for CKD-T2DM were potentially attributable to high BMI, with corresponding a significant increase of 29.97% (95% UI, 20.25 to 41.86) and 31.05% (95 % UI, 21.40 to 43.28). Details are presented in Table S1.

**TABLE S1:** Overall trends in age-standardized rate of chronic kidney disease due to diabetes mellitus type 2 attributable to low physical activity and high body-mass index

	Deaths rate in 2021	Percentage change in deaths rate, 1990–2021	PAF of deaths rate, 2021	Percentage change in PAF, 1990–2021	DALYs rate in 2021	Percentage change in DALYs rate, 1990–2021	PAF of DALYs rate, 2021	Percentage change in PAF, 1990–2021

Deaths and DALYs attributable to low physical activity								
Global	0.38 (0.15 to 0.63)	12.72% (-2.23% to 26.05%)	6.55% (2.71% to 10.72%)	-18.30% (-24.44% to -11.47%)	8.19 (3.21 to 13.60)	3.94% (-7.71% to 15.26%)	6.25% (2.51% to 10.22%)	-16.29% (-22.34% to -9.36%)

Central Asia	0.08 (0.03 to 0.13)	58.98% (12.82% to 132.15%)	4.31% (1.77% to 7.11%)	-42.84% (-53.18% to -25.32%)	2.91 (1.13 to 5.26)	-7.50% (-24.71% to 23.40%)	4.31% (1.71% to 7.28%)	-39.53% (-49.26% to -22.96%)

Eastern Europe	0.06 (0.02 to 0.10)	90.06% (47.46% to 151.62%)	7.61% (3.01% to 12.58%)	-21.72% (-37.61% to 1.24%)	1.93 (0.71 to 3.31)	0.91% (-18.06% to 27.62%)	7.25% (2.86% to 11.99%)	-19.71% (-32.54% to -1.28%)

Central Europe	0.07 (0.02 to 0.11)	-31.20% (-40.14% to -19.90%)	5.71% (2.39% to 9.22%)	-34.74% (-41.30% to -27.33%)	2.04 (0.84 to 3.49)	-31.25% (-39.03% to -21.26%)	6.10% (2.57% to 9.80%)	-29.85% (-35.84% to -22.50%)

Wetern Europe	0.15 (0.06 to 0.27)	-7.71% (-21.46% to 6.82%)	8.52% (3.62% to 13.91%)	-29.28% (-36.80% to -20.14%)	3.35 (1.34 to 5.67)	-22.08% (-30.90% to -11.71%)	8.20% (3.42% to 13.35%)	-26.18% (-32.93% to -17.97%)

High-income Asia Pacific	0.35 (0.14 to 0.62)	-24.05% (-37.21% to -8.50%)	9.67% (3.94% to 16.11%)	-7.03% (-21.31% to 9.05%)	6.93 (2.72 to 11.72)	-24.34% (-34.19% to -13.47%)	9.22% (3.63% to 15.34%)	-6.67% (-17.52% to 5.39%)

High-income North America	0.48 (0.20 to 0.78)	115.24% (73.16% to 173.87%)	6.00% (2.51% to 9.68%)	-40.13% (-48.91% to -28.43%)	10.37 (4.22 to 17.08)	79.10% (47.97% to 120.49%)	5.96% (2.44% to 9.62%)	-33.27% (-42.90% to -20.59%)

Australasia	0.09 (0.03 to 0.17)	33.48% (9.92% to 63.98%)	11.14% (4.63% to 18.28%)	-17.69% (-28.54% to -3.81%)	2.93 (1.22 to 5.05)	10.57% (-6.03% to 29.25%)	12.44% (5.16% to 20.98%)	-14.25% (-22.99% to -3.77%)

Oceania	0.70 (0.29 to 1.20)	11.34% (-27.49% to 65.64%)	5.17% (2.17% to 8.61%)	-13.09% (-25.59% to 1.98%)	16.00 (6.24 to 26.74)	3.84% (-28.92% to 51.72%)	5.17% (2.17% to 8.70%)	-16.57% (-27.87% to -3.84%)

Southeast Asia	0.45 (0.17 to 0.78)	4.67% (-16.69% to 25.16%)	4.35% (1.76% to 7.31%)	-20.66% (-29.45% to -9.49%)	10.31 (3.83 to 18.23)	2.91% (-15.12% to 21.78%)	4.33% (1.72% to 7.55%)	-16.91% (-25.24% to -6.81%)

South Asia	0.28 (0.11 to 0.49)	-4.90% (-25.49% to 20.56%)	5.32% (2.13% to 8.98%)	-26.19% (-36.79% to -13.25%)	6.88 (2.61 to 12.10)	-7.37% (-26.29% to 14.27%)	5.12% (2.03% to 8.63%)	-24.32% (-35.14% to -11.22%)

East Asia	0.47 (0.18 to 0.81)	-14.62% (-35.62% to 10.61%)	8.12% (3.37% to 13.88%)	2.36% (-13.47% to 21.33%)	9.27 (3.64 to 15.75)	-16.44% (-35.63% to 7.01%)	7.39% (2.96% to 12.63%)	5.60% (-10.52% to 23.49%)

Andean Latin America	0.86 (0.34 to 1.45)	-5.94% (-29.79% to 26.62%)	5.79% (2.32% to 9.61%)	-34.20% (-45.57% to -18.68%)	17.77 (7.19 to 30.48)	-0.80% (-24.81% to 32.67%)	6.21% (2.45% to 10.43%)	-27.33% (-39.59% to -11.13%)

Caribbean	0.71 (0.30 to 1.18)	16.99% (-5.71% to 37.86%)	6.22% (2.61% to 10.07%)	-18.28% (-29.09% to -7.74%)	15.57 (6.53 to 25.95)	17.68% (-2.81% to 37.41%)	6.41% (2.66% to 10.45%)	-16.09% (-25.88% to -6.17%)

Central Latin America	0.49 (0.20 to 0.84)	18.56% (-2.42% to 40.29%)	4.90% (2.02% to 8.20%)	-20.82% (-31.77% to -7.61%)	11.61 (4.83 to 19.67)	25.79% (5.07% to 47.81%)	4.99% (2.09% to 8.43%)	-19.65% (-30.75% to -8.02%)

Tropical Latin America	0.65 (0.26 to 1.11)	1.46% (-12.19% to 18.87%)	9.05% (3.64% to 15.08%)	-12.86% (-23.21% to 1.46%)	14.14 (5.76 to 24.12)	-2.56% (-15.39% to 13.63%)	9.08% (3.63% to 15.03%)	-10.30% (-21.33% to 4.37%)

Southern Latin America	0.31 (0.12 to 0.55)	-33.74% (-50.69% to -13.77%)	6.08% (2.41% to 10.48%)	-31.75% (-48.66% to -12.16%)	5.94 (2.46 to 10.42)	-36.31% (-50.52% to -17.19%)	5.98% (2.46% to 10.19%)	-30.50% (-46.09% to -10.61%)

North Africa and Middle East	0.50 (0.20 to 0.84)	-23.12% (-50.84% to 0.52%)	6.08% (2.49% to 9.89%)	-40.72% (-46.92% to -32.56%)	11.18 (4.55 to 18.93)	-22.33% (-47.39% to -0.48%)	6.58% (2.68% to 10.93%)	-36.84% (-43.68% to -27.03%)

Central Sub-Saharan Africa	0.60 (0.22 to 1.14)	-22.70% (-47.17% to 6.60%)	6.65% (2.74% to 11.18%)	-26.51% (-40.19% to -10.33%)	12.81 (4.87 to 23.91)	-23.85% (-45.69% to 2.03%)	6.52% (2.60% to 10.81%)	-25.85% (-39.53% to -11.15%)

Eastern Sub-Saharan Africa	0.50 (0.17 to 0.90)	-2.57% (-21.68% to 19.85%)	4.13% (1.56% to 7.52%)	2.31% (-12.42% to 20.63%)	9.29 (3.28 to 16.66)	-9.56% (-25.89% to 10.14%)	4.03% (1.52% to 7.16%)	4.48% (-9.27% to 21.68%)

Southern Sub-Saharan Africa	0.27 (0.11 to 0.48)	1.02% (-25.18% to 24.96%)	6.21% (2.60% to 10.04%)	-32.59% (-41.52% to -23.18%)	7.53 (3.03 to 13.43)	-6.95% (-25.96% to 12.44%)	6.92% (2.81% to 11.16%)	-32.20% (-41.03% to -21.55%)

Western Sub-Saharan Africa	0.32 (0.12 to 0.57)	-18.99% (-35.49% to -1.56%)	5.87% (2.39% to 9.82%)	-27.38% (-35.78% to -17.04%)	7.82 (3.10 to 13.59)	-18.27% (-31.46% to -1.63%)	6.29% (2.56% to 10.66%)	-24.15% (-32.86% to -12.34%)

**Deaths and DALYs attributable to high body-mass index**								

Global	2.07 (0.91 to 3.47)	79.26% (55.99% to 98.31%)	36.18% (15.97% to 57.93%)	29.97% (20.25% to 41.86%)	50.14 (22.56 to 79.15)	62.66% (44.24% to 78.73%)	38.24% (17.54% to 58.90%)	31.05% (21.40% to 43.28%)

Central Asia	0.94 (0.48 to 1.45)	138.99% (73.64% to 227.28%)	53.72% (26.60% to 72.38%)	-14.00% (-29.99% to 7.49%)	39.17 (21.27 to 55.11)	41.21% (14.80% to 75.87%)	58.11% (30.66% to 74.08%)	-7.63% (-21.41% to 9.30%)

Eastern Europe	0.51 (0.26 to 0.71)	155.85% (117.40% to 208.75%)	66.52% (36.01% to 79.69%)	5.47% (-5.15% to 21.28%)	18.41 (10.32 to 24.60)	33.99% (17.35% to 60.37%)	69.04% (39.51% to 80.56%)	6.62% (-2.45% to 20.96%)

Central Europe	0.62 (0.31 to 0.99)	-5.53% (-20.11% to 14.27%)	54.14% (27.79% to 76.78%)	-10.50% (-19.03% to 7.80%)	19.72 (10.44 to 28.06)	-10.25% (-20.86% to 6.01%)	59.01% (32.00% to 77.73%)	-8.45% (-16.01% to 6.59%)

Wetern Europe	0.91 (0.39 to 1.41)	23.05% (5.60% to 44.26%)	50.01% (24.28% to 72.34%)	-5.95% (-13.82% to 5.06%)	21.57 (10.00 to 31.71)	1.07% (-9.28% to 14.97%)	52.79% (25.43% to 72.54%)	-4.33% (-11.50% to 5.47%)

High-income Asia Pacific	1.13 (0.49 to 1.98)	-8.52% (-19.44% to 2.90%)	31.22% (15.56% to 52.93%)	11.74% (-0.46% to 24.26%)	25.17 (11.56 to 43.11)	-8.56% (-16.95% to -0.06%)	33.41% (17.04% to 56.33%)	12.64% (3.74% to 22.98%)

High-income North America	4.05 (1.86 to 6.40)	214.78% (157.14% to 297.75%)	50.02% (24.08% to 77.06%)	-12.61% (-23.77% to 4.89%)	94.03 (44.88 to 138.06)	143.53% (105.27% to 189.07%)	54.00% (27.08% to 77.94%)	-9.31% (-20.26% to 3.91%)

Australasia	0.53 (0.27 to 0.78)	67.28% (42.30% to 96.00%)	64.50% (35.68% to 82.38%)	2.86% (-6.12% to 15.83%)	15.98 (8.70 to 21.88)	31.47% (15.48% to 51.29%)	67.74% (39.59% to 82.26%)	1.88% (-5.49% to 12.60%)

Oceania	4.57 (1.76 to 8.27)	53.45% (1.65% to 128.70%)	33.50% (14.24% to 58.05%)	19.68% (4.48% to 45.79%)	109.87 (43.34 to 192.60)	41.45% (-1.82% to 103.49%)	35.42% (14.81% to 60.61%)	13.48% (1.39% to 35.68%)

Southeast Asia	1.67 (0.59 to 3.62)	110.91% (64.89% to 158.15%)	16.10% (5.75% to 31.91%)	59.79% (39.91% to 86.77%)	44.54 (15.93 to 93.97)	102.56% (65.48% to 141.15%)	18.73% (6.76% to 37.66%)	63.53% (45.74% to 87.09%)

South Asia	1.12 (0.48 to 2.03)	99.73% (51.32% to 159.83%)	21.34% (9.40% to 38.49%)	55.03% (32.41% to 87.27%)	32.31 (14.05 to 56.50)	98.32% (57.68% to 150.33%)	24.05% (10.59% to 41.36%)	62.05% (40.92% to 94.52%)

East Asia	2.00 (0.81 to 3.67)	50.57% (14.70% to 93.20%)	34.25% (13.42% to 60.47%)	80.58% (51.28% to 115.13%)	45.95 (18.54 to 82.57)	46.48% (14.52% to 86.06%)	36.57% (14.71% to 63.83%)	85.13% (52.15% to 123.95%)

Andean Latin America	6.90 (2.93 to 11.19)	57.36% (21.82% to 119.31%)	46.36% (20.10% to 69.49%)	10.02% (-5.65% to 45.15%)	147.40 (65.40 to 228.82)	52.02% (17.38% to 107.84%)	51.50% (24.03% to 70.83%)	11.29% (-3.77% to 45.45%)

Caribbean	4.85 (2.02 to 7.98)	88.80% (60.12% to 126.43%)	42.52% (20.56% to 67.20%)	31.84% (19.54% to 49.45%)	112.51 (49.26 to 172.98)	81.25% (55.40% to 116.72%)	46.29% (23.04% to 67.13%)	29.21% (17.57% to 47.03%)

Central Latin America	5.06 (2.39 to 8.00)	91.34% (66.59% to 123.69%)	50.31% (23.83% to 75.52%)	27.72% (15.78% to 44.31%)	125.48 (62.43 to 190.67)	90.39% (64.84% to 125.01%)	54.00% (26.62% to 75.92%)	21.64% (11.06% to 38.69%)

Tropical Latin America	3.77 (1.72 to 5.68)	43.16% (24.92% to 66.16%)	52.09% (24.73% to 74.56%)	22.96% (7.64% to 42.55%)	85.51 (40.51 to 122.33)	29.04% (14.87% to 49.95%)	54.84% (27.25% to 74.76%)	18.84% (7.27% to 37.27%)

Southern Latin America	2.81 (1.29 to 4.26)	-6.41% (-21.17% to 16.62%)	55.74% (27.27% to 77.83%)	-3.72% (-16.31% to 16.92%)	58.73 (28.84 to 84.13)	-10.32% (-22.38% to 9.01%)	59.03% (30.15% to 77.83%)	-2.22% (-13.98% to 17.50%)

North Africa and Middle East	4.17 (1.85 to 6.63)	33.67% (-13.79% to 71.94%)	50.91% (23.89% to 73.71%)	3.10% (-9.36% to 17.01%)	93.06 (44.34 to 140.97)	28.82% (-11.86% to 62.52%)	54.71% (27.35% to 74.47%)	4.85% (-6.84% to 20.16%)

Central Sub-Saharan Africa	2.92 (1.04 to 5.64)	47.84% (4.51% to 104.23%)	32.25% (12.93% to 57.64%)	40.75% (17.82% to 65.31%)	70.05 (26.82 to 129.83)	42.28% (2.12% to 92.13%)	35.64% (14.60% to 59.81%)	38.72% (17.83% to 62.52%)

Eastern Sub-Saharan Africa	2.26 (0.83 to 4.37)	60.14% (29.03% to 106.59%)	18.76% (7.39% to 33.65%)	67.78% (42.28% to 108.56%)	49.93 (18.52 to 92.38)	52.39% (22.34% to 99.25%)	21.64% (8.72% to 38.37%)	75.70% (47.15% to 119.10%)

Southern Sub-Saharan Africa	1.89 (0.73 to 3.34)	86.36% (35.24% to 128.13%)	43.49% (17.30% to 68.89%)	24.51% (8.40% to 41.50%)	52.65 (23.54 to 84.77)	56.63% (27.31% to 85.51%)	48.42% (21.81% to 69.80%)	14.29% (0.18% to 29.44%)

Western Sub-Saharan Africa	1.97 (0.82 to 3.47)	34.89% (8.35% to 76.63%)	35.96% (15.28% to 57.42%)	20.87% (7.54% to 48.79%)	50.51 (23.27 to 80.56)	29.69% (7.05% to 67.97%)	40.69% (17.96% to 59.12%)	20.35% (6.90% to 50.26%)


PAF, population attributable fractions; DALYs, disability-adjusted life years

Across the 21 GBD regions, the highest low-PA-related ASR of deaths and DALYs for CKD-T2DM in 2021 were 0.86 (95% UI, 0.34 to 1.45) per 100,000 people and 17.77 (95% UI, 7.19 to 30.48) per 100,000 people, respectively, both seen in Andean Latin America. Meanwhile, Eastern Europe had the lowest ASR of deaths and DALYs for CKD-T2DM attributable to low PA, with values of 0.06 (95% UI, 0.02 to 0.10) per 100,000 people and 1.93 (95% UI, 0.71 to 3.31) per 100,000 people, respectively. Between 1990 and 2021, the largest increase of low-PA-related ASR of deaths and DALYs for CKD-T2DM was observed in High-income North America, with values of 115.23% (95% UI, 73.16 to 173.87) and 79.10% (95% UI, 47.97 to 120.49), respectively. In contrast, Southern Latin America had the largest decrease of low-PA-related ASR of deaths (-33.73% [95% UI, -13.72 to -50.69]) and DALYs (-36.32% [95% UI, -17.19 to -50.52]) for CKD-T2DM since 1990. In 2021, the highest high-BMI-related ASR of deaths and DALYs for CKD-T2DM were 6.90 (95% UI, 2.93 to 11.19) and 147 (95% UI, 65.40 to 228.82) per 100,000 people, respectively, also seen in Andean Latin America. Eastern Europe had the lowest ASR of deaths (0.51 [95% UI, 0.26 to 0.71] per 100,000 people) and DALYs (18.40 [95% UI, 10.32 to 24.60] per 100,000 people) for CKD-T2DM in 2021 again. Similarly, High-income North America had the largest increase of high-BMI-related ASR of deaths (214.78% [95% UI, 157.14 to 297.75]) and DALYs (143.53% [95% UI, 105.27 to 143.53]) for CKD-T2DM between 1990 and 2021. The largest reduction in high-BMI-related ASR of deaths and DALYs for CKD-T2DM from 1990 to 2021 occurred in High-income Asia Pacific (-8.52% [95% UI, -19.44 to 2.90]) and Southern Latin America (-10.32% [95% UI, -22.38 to 9.01]), respectively. Details are shown in Table S1.

There were differences in the disease burden of CKD-T2DM attributable to low PA between females and males across 21 GBD regions. For ASR of DALYs, the female to male ratio of low-PA-related CKD-T2DM ranged from 0.74 in Southern Sub-Saharan Africa to 1.57 in Eastern Europe. A similar tendency was observed for high-BMI-related CKD-T2DM, with the female to male ratio ranging from 0.70 in High-income Asia Pacific to 1.31 in Eastern Europe. For PAL of age-standardized DALYs, there was an excess female CKD-T2DM risk across all GBD regions, with the global female to male ratio of low-PA-attributable CKD-T2DM exceeded 1.32 and that of high-BMI-attributable CKD-T2DM exceed 1.26. Details are shown in Figures S1 and S2.

### Temporal patterns in chronic kidney disease due to diabetes mellitus type 2 attributable to low physical activity and high body-mass index by SDI regions

**FIG. 1:**
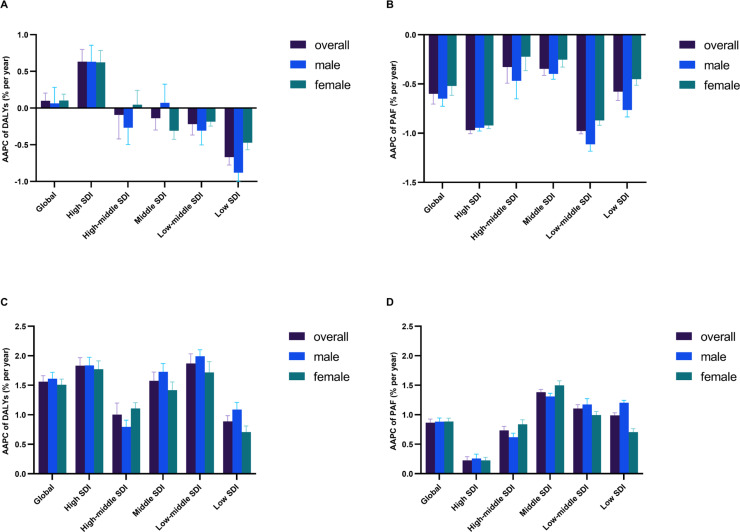
Average annual percent change (AAPC) of age-standardized DALYs rate for CKD-T2DM attributable to low physical activity from 1990 to 2021 in A, and AAPC of population attributable fractions (PAF) of age-standardized DALYs for CKD-T2DM attributable to low physical activity from 1990 to 2021 in B, and AAPC of age-standardized DALYs rate for CKD-T2DM attributable to high body-mass index from 1990 to 2021 in C, and AAPC of PAF of age-standardized DALYs for CKD-T2DM attributable to high body-mass index from 1990 to 2021 in D. DALYs = disability-adjusted life years, CKD-T2DM = Chronic kidney disease due to diabetes mellitus type 2, SDI = socio-demographic index.

**FIG. 2:**
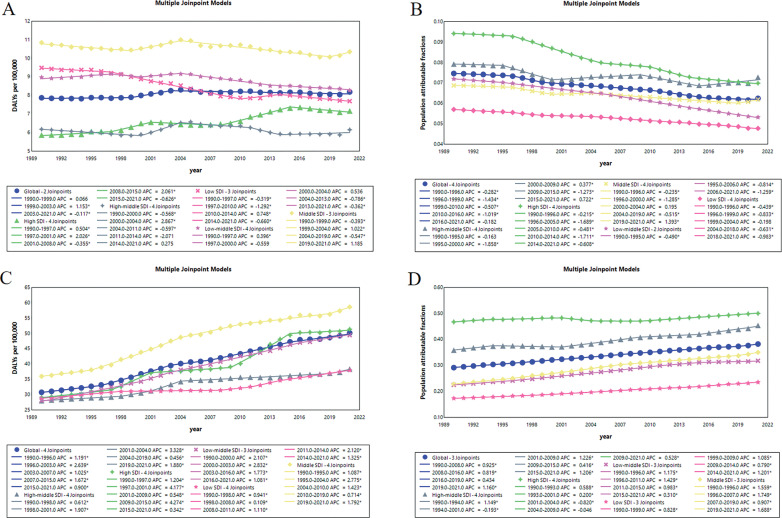
Temporal variation trends in age-standardized DALYs rate for low-PA-related CKD-T2DM in A, and proportion of age-standardized DALYs for CKD-T2DM attributable to low PA on total CKD-T2DM in B, and corresponding trends for high-BMI-related CKD-T2DM burden in C and D, respectively. DALYs = disability-adjusted life years, PA = physical activity, CKD-T2DM = chronic kidney disease due to diabetes mellitus type 2, BMI = body-mass index.

Globally, the ASR of DALYs for CKD-T2DM attributable to low PA changed slightly between 1990 and 2021, from 7.88 (95% UI, 3.07 to 13.01) to 8.18 (95% UI, 3.21 to 13.60) per 100,000 people with an AAPC of 0.09% (95% UI, -0.01 to 0.20) (Figure 1A). The tendency increased substantially between 1999 and 2003 with a segmental AAPC of 1.15% (95% CI, 0.38 to 1.93), and then experienced a slight decline with a segmental AAPC of -0.12% (95% CI, -0.17 to -0.07) (Figure 2A). The high SDI regions observed a solely marked increase from 5.88 (95% UI, 2.28 to 9.62) to 7.17 (95% UI, 2.96 to 11.76) per 100,000 people between 1990 and 2021 with an AAPC of 0.63% (95% CI, 0.47 to 0.80), whereas middle SDI regions had the most severe CKD-T2DM burden from 10.84 (95% UI, 4.28 to 18.05) to 10.35 (95% UI, 4.04 to 17.29) per 100,000 people through the past 31 years (Figure 1A, Figure 2A). Between 1990 and 2021, the percentage of global age-standardized DALYs for CKD-T2DM attributable to low PA fell from 7.46% (95% UI, 2.96 to 12.41) to 6.24% (95% UI, 2.51 to 10.22), with an AAPC of -0.60% (95% CI, -0.70 to -0.49) (Figure 1B). While declines occurred across all region by SDI, the highest reductions were observed in low-middle SDI region from 7.20% (95% UI, 2.83 to 12.21) to 5.32% (95% UI, 2.16 to 8.90) with a AAPC of -0.98 (95% CI, -1.01 to -0.95) (Figure 1B, Figure 2B). For high-BMI-related CKD-T2DM burden, the global ASR of DALYs surged from 30.82 (95% UI, 13.87 to 49.54) to 50.14 (95% UI, 22.56 to 79.15) per 100,000 people between 1990 and 2021, with an AAPC of 1.56% (95% CI, 1.46 to 1.66) (Figure 1C). The highest segmental AAPC occurred during 1996 to 2003 (2.64% [95% CI, 2.45 to 2.83]) and 2007 to 2015 (1.67% [95% CI, 1.52 to 1.82]) (Figure 2C). By SDI, the ASR of DALYs increase across all regions. The low-middle SDI regions had the highest increment of CKD-T2DM burden with an AAPC of 1.87% (95% CI, 1.70 to 2.03), while the middle SDI region experienced the most severe CKD-T2DM burden from 36.03 (95% UI, 15.18 to 65.02) to 58.59 (95% UI, 25.50 to 98.29) per 100,000 people since 1990 (Figure 1C, Figure 2C). In contrast to low-PA-related CKD-T2DM, the percentage of global age-standardized DALYs for CKD-T2DM attributable to high BMI increased substantially from 29.18% (95% UI, 12.83 to 46.17) to 38.23% (95% UI, 17.54 to 58.90) with an AAPC of 0.87% (95% CI, 0.83 to 0.93) since 1990 (Figure 1D, Figure 2D). The middle SDI regions had the largest increment of population attributable fractions of high-BMI-related CKD-T2DM burden with an AAPC of 1.38% (95% CI, 1.34 to 1.43), while high SDI regions experienced the highest percentage of CKD-T2DM burden attributable to high BMI from 46.71% (95% UI, 22.39 to 64.26) to 49.98% (95% UI, 24.73 to 71.69) (Figure 1D, Figure 2D)

### Age-period-cohort patterns in chronic kidney disease due to diabetes mellitus type 2 attributable to low physical activity and high body-mass index

**FIG. 3:**
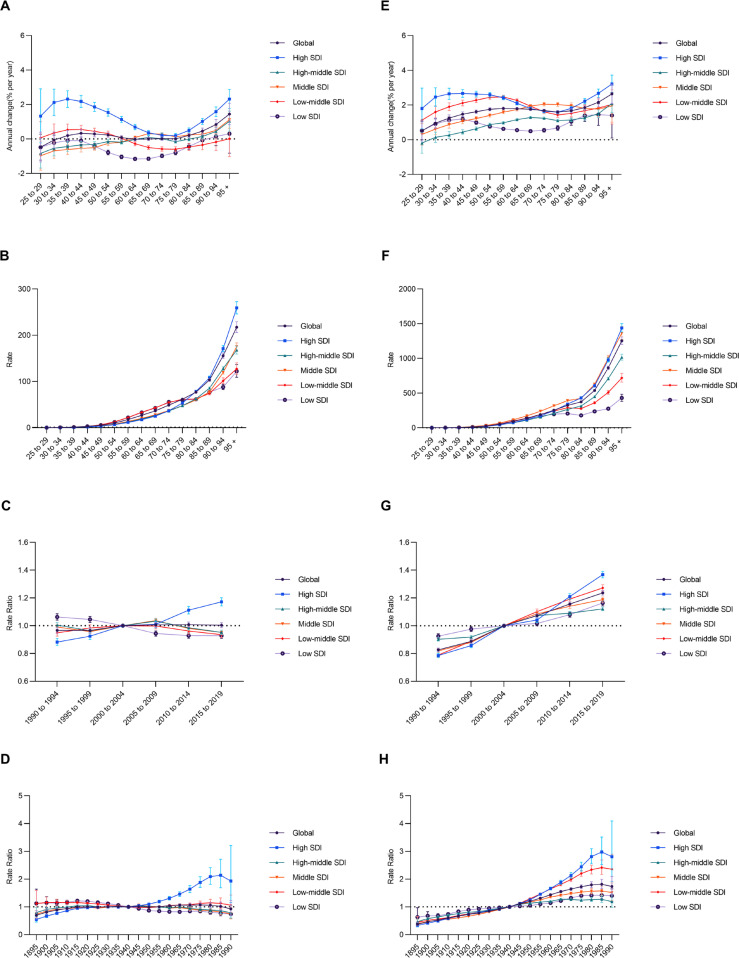
Age-period-cohort patterns of DALYs rate for chronic kidney disease due to diabetes mellitus type 2 attributable to low physical activity and high body-mass index. Local drift of annual percent change across age groups in A, age effects in B, period effects in C, cohort effects in D, and those of high body-mass index in E to H, respectively. DALYs = disability-adjusted life years, SDI = socio-demographic index.

The local drift of DALYs derived from age-period-cohort model, which means the annual percent change (APC) in DALYs rate of low-PA-related CKD-T2DM for each age group. Global CKD-T2DM burden attributable to low PA demonstrated a surging trend from 25 to 39 years old and 75 to 95+ years old, respectively. Specifically, the high SDI region had the largest APC of low-PA-related DALYs for CKD-T2DM across all age groups, with the highest APC occurred in 35–39 age group (2.31% [95% CI, 1.83 to 2.80]). In all SDI regions, the APC of low PA attributable DALYs rate for CKD-T2DM maintained a gradually weakened upward trend in age groups younger than 40 and showed a gradually increased upward trend in age groups older than 75 (Figure 3A). Similarly, the APC of global DALYs rate for high-BMI-related CKD-T2DM maintained a relatively stable upward trend across all age groups. The upward trend occurred in age groups younger than 44 across all SDI regions, while most SDI regions experienced a surging upward trend in age groups older than 75 except middle SDI region. The high SDI region had the largest APC in most age groups except 60–84, with the upward trend gradually weakened in age groups younger than 44 and slightly increased in age groups older than 75 (Figure 3E).

The age effects pattern was similar over SDI regions, with the DALYs rate for low-PA-related CKD-T2DM and high-BMI-related CKD-T2DM increasing with age, especially for elderly. Generally, DALYs rate for CKD-T2DM attributable to low PA was higher across age groups less than 75 in low SDI and low-middle SDI regions compared with other SDI regions, while that of high BMI was higher for most age groups in middle SDI region (Figure 3B and 3F). Regarding period effects, there was globally a flat trend for DALYs rate of low-PA-related CKD-T2DM from1990 (Rate Ratio = 0.97 [95% CI, 0.95 to 0.99]) to 2019 (Rate Ratio = 1.00 [95% CI, 0.99 to 1.02]), with the risk increasing with period in high SDI region and decreasing with period in low SDI region. In contrast, there was a stable upward trend for DALYs rate of high-BMI-attributed CKD-T2DM from 1990 (Rate Ratio = 0.83 [95% CI, 0.81 to 0.84]) to 2019 (Rate Ratio = 1.24 [95% CI, 1.22 to 1.25]), with the largest increment occurred in high SDI region (Rate Ratio = 0.79 [95% CI, 0.77 to 0.80]; Rate Ratio = 1.37 [95% CI, 1.34 to 1.39], respectively) (Figure 3C and 3G). For birth cohort effects, DALYs rate for CKD-T2DM attributable these two modifiable behavioral risk factors generally showed a trend similar to that of period effects (Figure 3D and 3H).

### Early-onset chronic kidney disease due to diabetes mellitus type 2 attributable to low physical activity and high body-mass index

In 2021, most of DALYs for CKD-T2DM among people younger than 40 was attributed to low PA and high BMI across all SDI regions (Figure S3). For instance, low PA and high BMI contributed to 14.27% (95% UI, 5.06 to 26.61) and 73.94% (95% UI, 60.64 to 78.26) DALYs for overall CKD-T2DM at 25–29 age group in high SDI region, respectively, which were the largest PAL across all age groups (Figure S3). In other SDI regions, similar patterns for each age group were observed.

**FIG. 4:**
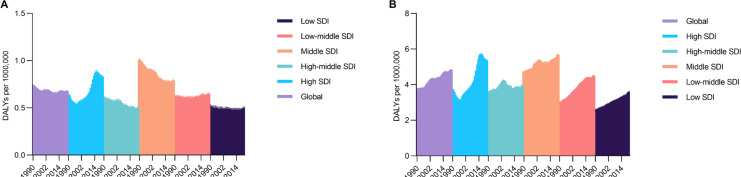
Age-standardized DALYs rate for early-onset chronic kidney disease due to diabetes mellitus type 2 attributable to low physical activity from 1990 to 2021 in A, and that of high body-mass index in B. DALYs = disability-adjusted life years.

There were also differences of ASR of DALYs for the early-onset CKD-T2DM attributable to low PA across SDI regions. Overall, the global early-onset CKD-T2DM exhibited a downward trend with an AAPC of -0.28% (95% CI, -0.51 to -0.05) since 1990, however, an opposite trend emerged with an AAPC of 0.23% (95% CI, 0.14 to 0.33) in last 5 years. The highest early-onset CKD-T2DM rates occurred in middle SDI region with an AAPC of -0.85% (95% CI, -0.98 to -0.71) throughout the past 32 years and an AAPC of -0.05% (95% CI, -0.21 to 0.12) in last 5 years. The high SDI and low-middle SDI regions showed a gradually increasing upward trend with an AAPC of 0.86% (95% CI, 0.73 to 0.99) and 0.11% (95% CI, 0.04 to 0.18) since 1990, respectively (Figure 4A). Regarding high-BMI related early-onset CKD-T2DM, there was a consistent increasing trend for ASR of DALYs across all SDI regions with a global AAPC of 0.78% (95% CI, 0.58 to 0.97) since 1990. There were marked increments were seen in high SDI, low SDI and low-middle SDI regions with an AAPC exceeding 1.1% throughout the past 32 years, while middle SDI region still had the most severe early-onset CKD-T2DM burden with the ASR of DALYs surging from 4.73 (95% UI, 4.66 to 4.80) to 5.69 (95% UI, 5.61 to 5.77) per 100,000 people between 1990 and 2021 (Figure 4B).

### Correlation between chronic kidney disease due to diabetes mellitus type 2 attributable to low physical activity and high body-mass index and Socio-demographic Index

**FIG. 5:**
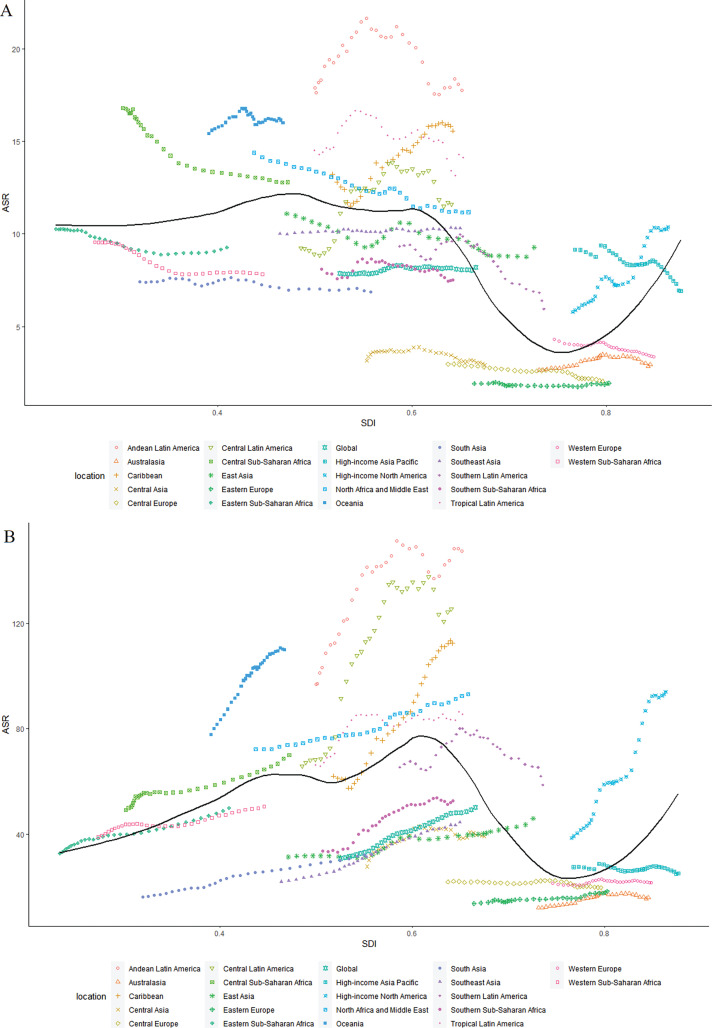
Age-standardized DALYs rate for CKD-T2DM attributable to low physical activity across 21GBD regions by Socio-demographic Index for both sexes combined from 1990 to 2021 in A, and that of high body-mass index from 1990 to 2021 in B. DALYs = disability-adjusted life years; CKD-T2DM = chronic kidney disease due to diabetes mellitus type 2.

From 1990 to 2021, the relationship between the ASR of DALYs for CKD-T2DM attributed to low PA and SDI was nonlinear across 21 GBD regions, showing a relatively stable trend before SDI ≈0.6 and then dramatically changing with higher SDI values (Figure 5A). Regarding high-BMI-related CKD-T2DM, there was also a nonlinear relationship between the ASR of DALYs and SDI, showing a relatively stable upward trend before SDI ≈0.6 and, a similar trend occurred with the ASR of DALYs declining with high-middle SDI value and then surging with higher SDI value (Figure 5B).

**FIG. 6:**
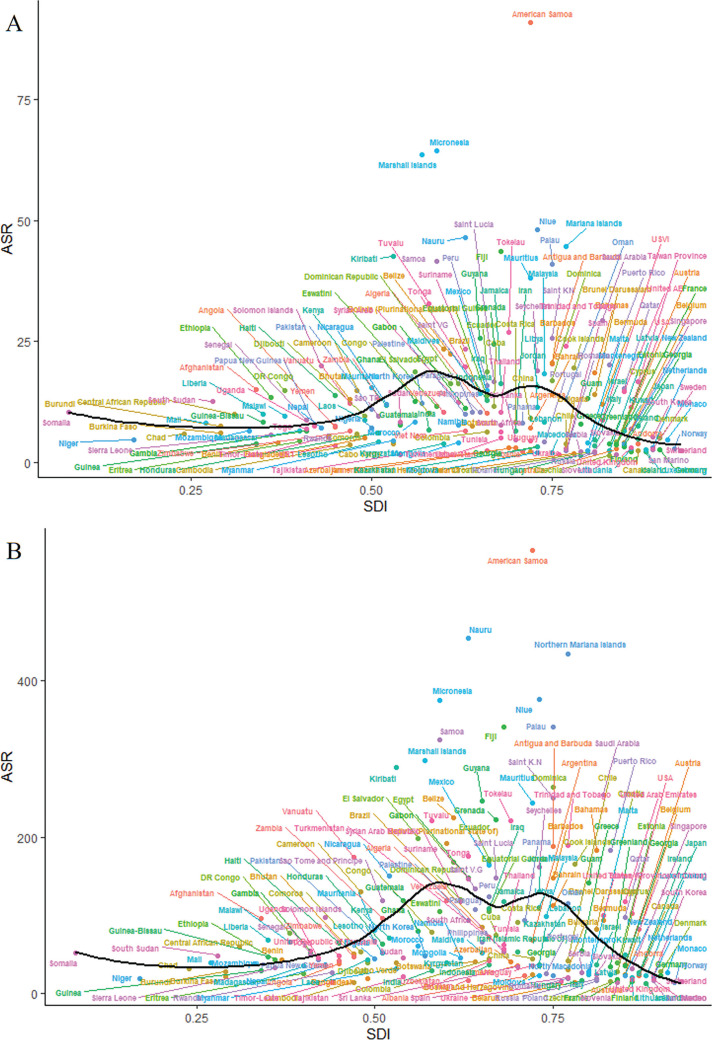
Age-standardized DALYs rate for CKD-T2DM attributable to low physical activity across 204 countries and territories by Socio-demographic Index for both sexes combined in 2021 in A, and that of high body-mass index in 2021 in B. DALYs = disability-adjusted life years; CKD-T2DM = chronic kidney disease due to diabetes mellitus type 2.

For 204 countries and territories, the relationship between ASR of DALYs for low-PA-attributed CKD-T2DM and SDI was also nonlinear, reaching a peak at a SDI value of 0.6 before gradually decreasing with higher SDI value (Figure 6A). A highly similar trend was observed between the ASR of DALYs for high-BMI-attributed CKD-T2DM and SDI (Figure 6B).

### Future forecasts of global chronic kidney disease burden

Our forecasts indicated a generally surging trend in ASR of DALYs for CKD-T2DM across 21 GBD regions. Between 2021 and 2050, the global ASR of DALYs for CKD-T2DM projected to rise from 131.08 (95% UI, 112.75 to 152.49) to 166.91 (95% UI, 122.01 to 221.45) per 100,000 people. Low-middle SDI and middle SDI regions (e.g. Andean Latin America, Caribbean and Oceania) projected to experience the most severe CKD-T2DM burden in future, while the high SDI region (e.g. West Europe, Central Europe and Australasia) is predicted to experience little change in its CKD-T2DM burden until 2050 (Figure S4).

In 2050, the global low PA exposure may have a slightly increase since 2021, however, there are marked differences across regions. Tropical Latin America, North Africa and Middle East may have the largest low PA risk across regions between 2021 and 2050, while Australasia is projected to show with low PA exposure rising from 6.97 (95% UI, 3.55 to 13.77) to 10.21 (95% UI, 5.25 to 19.38) per 100,000 people (Figure S4). For high BMI exposure, there is overwhelmingly a rising trend across 21 GBD regions. Globally, the high BMI risk projected to increase from 16.48 (95% UI, 14.10 to 19.31) to 22.69 (95% UI, 18.50 to 26.91) per 100,000 people between 2021 and 2050. Similarly, Central Latin America and Australasia show the largest high BMI risk until 2050, while North Africa and Middle East have the most severe obesity risk with the high BMI exposure surging from 28.01 (95% UI, 23.19 to 32.42) to 39.49 (95% UI, 30.42 to 46.55) per 100,000 people (Figure S4).

## DISCUSSION

Strong evidence has revealed that low PA and high BMI exposure lead to a major public health challenge globally, while the present study comprehensively offers an updated knowledge of the substantial CKD-T2DM burden attributable to these two most modifiable lifestyle risk factors. We generally illuminate the trends in CKD-T2DM burden associated with low PA and high BMI across 21 GBD regions between 1990 and 2021, and suggest diverse patterns in CKD-T2DM attributed to these risk factors by SDI regions throughout the past 32 years. The temporal variations of CKD-T2DM burden attributed to low PA and high BMI were estimated using joinpoint model, and the effects of epidemiological shift were detected by age-period-cohort. This study predicted future forecasts of global CKD-T2DM burden and, corresponding low PA and high BMI exposure until 2050 were conducted using GBD Foresight Tool.

Globally, the ASR of deaths and DALYs for CKD-T2DM attributable to low PA maintains a relatively stable trend since 1990, while the proportion of CKD-T2DM burden due to low PA show a decreasing trend. In contrast, the ASR of deaths and DALYs for high-BMI-related CKD-T2DM show a marked increase between 1990 and 2021. Similar trends were found in PAF of CKD-T2DM burden attributable to high BMI across all SDI regions. These findings may suggest an imbalance of improvement in global lifestyle intervention to overcome noncommunicable disease like chronic kidney disease consistent with previous studies [22, 23]. In addition, results from the joinpoint model found that regional differences and temporal variations substantially underlie the overall reductions. For instance, the ASR of DALYs for low-PA-attributed CKD-T2DM in low SDI, low-middle SDI, middle SDI and high-middle SDI regions experience reductions in past three decades, whereas that of low-PA-related CKD-T2DM in high SDI undergoes substantial increases with an AAPC of 0.63. With respect to temporal patterns, the low-PA-related CKD-T2DM burden in high SDI region presents an increasing trend in past 3 decades, however, jointpoint analysis revealed a decreasing trend with a segmental AAPC of -0.63% during 2015 and 2021. In middle SDI region, low-PA-related CKD-T2DM burden generally fell from 10.84 to 10.35 per 100,000 people between 1990 and 2021, however, there was notably a substantial increase with segmental AAPC exceeding 1.2% in past two years. Such a temporal inconsistency occurred for high-BMI-related CKD-T2DM burden. While the AAPC of high-BMI-related CKD-T2DM reached a marked value of 1.83%, there was a lowest segmental AAPC during past five years in high SDI region. In contrast, the low SDI, low-middle SDI and middle SDI regions underwent the largest increase of high-BMI-related CKD-T2DM burden in the last 5 years with segmental AAPC substantially exceeding 1%. All these findings suggest that the use of global AAPC in full range of period may not comprehensively assess the CKD-T2DM burden due to the presence of regional and temporal variations, increased focus should be concentrated on an updated temporal transition of CKD-T2DM burden especially in underdeveloped areas [24, 25].

Age-period-cohort analyses reveal that both DALYs rate attributable to low PA and high BMI demonstrate a marked increasing trend with age globally. Period effects show a relatively flat trajectory for low-PA-related CKD-T2DM DALYs rate except for the high SDI region that exhibiting an upward trend throughout past three decades. In contrast, high-BMI-related CKD-T2DM DALYs rate exhibits a sound surging trend across all SDI regions, while high SDI region undergoes the largest increment again. Similar patterns are seen for cohort effect, which means successive improvements for low-PA-related CKD-T2DM burden and an inadequate effort for obesity control. Notably, the local drift of percent change annually of DALYs rate for CKD-T2DM attributable to low PA and high BMI occurred for each age group across SDI regions and especially in high SDI regions. Present results found that people younger than 40 or older than 75 showed highest the increasing trend in DALYs rate for low-PA-related CKD-T2DM, and similar age patterns were observed for high-BMI-related CKD-T2DM burden. Notably, the early-onset CKD-T2DM attributable to these two modifiable risk factors deserve more focus. For example, the PAF of CKD-T2DM deaths and DALYs contributed to both these two risk factors reached a peak among population younger than 40. On the other hand, the APC of DALYs rate for CKD-T2DM attributable to low PA and high BMI even exceeding 2% at 30 to 40 age groups in high SDI region. This analyses further found that the ASR of DALYs for high-BMI-related CKD-T2DM in population younger than 40 experienced a surging trend across all five SDI regions. That of low-PA-related CKD-T2DM in these age groups showed a similar pattern in high SDI region and low-middle SDI region, and gradually weakened decrease in other SDI regions. These findings indicate that as the presence of variations for each age group, considering only about the net effect may not be enough to assess CKD-T2DM burden accurately [26, 27]. Findings from age-period-cohort insights offer substantial backing to the imbalance in curbing CKD-T2DM burden attributable to lifestyle risk factors, especially in middle SDI and high SDI regions. Although there is progressive higher influence of low PA exposure on elderly, which can be explained by the descending of physical performance and the ascending of disease vulnerability [27, 28]. However, a previous study has identified young-and-middle-aged people as the most attractive target populations for efforts to attenuate the onset of noncommunicable disease and then extend healthspan [29]. It may be beneficial to foster adherence to a physically active lifestyle paired with management of obesity in early life especially on the context that early-onset T2DM is an independent risk factor of end-stage renal disease [30–32]. To prevent DALYs of early-onset CKD-T2DM, this study suggests that more focus should be centered on individuals younger than 40 whom are vulnerable to CKD-T2DM attributable to low PA and high BMI.

Present analyses for the association between SDI and CKD-T2DM burden demonstrate a highly consistent trajectory in ASR of DALYs for CKD-T2DM attributable to low PA and high BMI with increasing SDI in 204 countries and territories. For those SDI < 0.6 regions, the upward trend may be explained by nutrition and lifestyle transition followed with economic development, such a shifting of dietary patterns and an inactive lifestyle may precipitate obesity incidence. Generally, the shift in dietary patterns shifting involved the accessibility, composition, and consumption of foods transitioning toward high-calorie diets like highly processed food, red meat, sugar-sweetened beverage, and animal-source products may occur in those lower SDI regions as national-level income grows [13]. However, due to the lack of corresponding optimization in the public health care system, these surging shift in dietary pattern paired with a physically inactive lifestyle may prompt an increase of metabolism disorder. This may boost chronic kidney disease risk factors like diabetes, hypertension, and hyperlipidemia. For those SDI > 0.7 countries and territories, as economies further developed, greater focus and resources are centered on strengthening health care system paired with education for public health awareness and governmental interventions aimed to deal with noncommunicable disease like T2DM and cardiovascular disease [13, 33]. In addition, improvement in the accessibility of medical screening, emergency services, and acute treatment coverage may facilitate earlier CKD-T2DM detection and intervention to ameliorate disease burden. All these systemic transition may comprehensively mitigate the CKD-T2DM burden due to low PA and high BMI in higher SDI areas. Specifically, analyses for the association between SDI and CKD-T2DM DALYs rate contributed to low PA and high BMI in 21 GBD regions since 1990 show an inconsistent trajectory in higher SDI interval (SDI > 0.75) with analyses for that in 204 countries and territories. The remarkable higher CKD-T2DM burden due to low PA and high BMI in High-income North America and that of low PA in High-income Asia Pacific may explain this inconsistence, which are outliers compared to the trend in other higher SDI regions.

The forecasts of CKD-T2DM burden paired with low PA and high BMI exposure across 21 GBD regions in future provide support to present analyses for the longitudinal patterns of CKD-T2DM contributed to these two lifestyle risk factors. Generally, low-middle SDI and middle SDI regions (e.g. Caribbean and Oceania) projected to experience the most severe CKD-T2DM burden until 2050, while the lowest CKD-T2DM burden predicted to occur in high SDI area again (e.g. West Europe and Australasia). For low PA and high BMI exposure, middle SDI region (e.g. North Africa and Middle East) projected to experience the largest risk in nearly future 30 years, whereas low SDI region (e.g. Sub-Saharan Africa and South Asia) demonstrates a relatively lower risk compared with other regions. The regional variations by SDI may reflect the inequality of overall public health resources due to national development difference according to recent study [13, 26]. For example, the future forecasts suggest that there are marked ascendancy in low PA and high BMI exposure in Australasia through 2050, however, the lowest CKD-T2DM burden in future three decades is also projected to occur in Australasia. Conversely, an inverse pattern is predicted to seen in Southeast Asia. This forecast in such a traditional developed country is consistent to results mentioned above for the lower CKD-T2DM burden due to low PA and high BMI observed in Australasia, which indicates a more robust public health care system to resist lifestyle risk.

Generally, the highly consistent trajectories for both observed low-PA-related and high-BMI-related CKD-T2DM burden with increasing SDI may be caused by mutualism of these two modifiable risk factors. Although there are improvements in curbing low-PA-related CKD-T2DM burden in most regions, however, the projected rising trend in high BMI exposure and subsequent chronic disease burden remains severe. It highlights an urgency for ameliorating overweight and obesity risk via taking multidimensional measures. For those regions with lower SDI (< 0.6), a need for the development, implementation and fostering of an anti-obesity combined with anti-inactivity action plan was urgent. Recent reports have suggested that even just 10% reductions of inactivity could prevent exceeding 533,000 overall deaths per year, while this value may rise to more than 1,300,000 by a 25% decrease [34]. For the purpose of achieving further control in CKD-T2DM burden caused by modifiable lifestyle factors, a targeted strategy of governmental interventions like marketing regulation, front-of-package labeling, and sugar-sweetened beverage taxation paired with corresponding health educational campaigns based on community and constructed environment facilitating physically active lifestyle should be taken [35]. Policies aimed at facilitating balanced diet patterns must be paired with strengthening equitable access to healthy foods, which involves the allowance of vegetables, fruits, and whole grains, with attempts concerning taxation of sugar-sweetened and ultra-processed foods [13]. Moreover, resources should be oriented toward creating active-favorable environments like public sport parks, bike lanes, sidewalks, paired with exercise encouragement based on community and school [36]. Efforts in the health care system should be taken to tackle social inequality hindering early intervention and control of CKD-T2DM risk factors via broadening screening coverage, lifestyle medicine counseling, and specifically supporting society work programs for health care of marginal populations [25]. Thus, dispersing appropriate guidelines for management of BMI and PA particularly in subgroups of population which are more vulnerable to lifestyle risk factors like low PA and high BMI exposure, is crucial.

There are certain limitations to our work. Because all data analyzed in the present study are from GBD, it depended on the quality of the methodology performed by GBD Collaborator. First, PA data used in GBD were primarily based on self-retrospective measures (e.g. IPAQ questionnaires), which were subject to biases. However, using these elementary tools make cross-country study feasible in the context that objective methods like accelerometer utilized to collect PA data in large scale population are only implemented in developed countries. Second, the GBD database included only CKD-T2DM burden due to low PA and high BMI in adult individuals, while it is well known that PA and BMI level differ for age strata. Third, we could only classify high BMI and low PA exposure with definitions from GBD, which may limit the universality of our results. Finally, we failed to explore more CKD-T2DM pattern due to lifestyle risk factors across several subgroups of populations (e.g. ethnic groups).

Given the observed regional disparities in the CKD-T2DM burden trends associated with high BMI and low PA, future research should prioritize disease pattern in both developing regions such as North Africa and Middle East, as well as developed nations in High-income North America. Furthermore, the rising trend in CKD-T2DM attributable to these modifiable factors among populations under 40 years old underscores the urgent need for a further investigation into early-onset CKD-T2DM. Enhanced attention should be directed toward populations that are vulnerable to lifestyle-related CKD-T2DM, as this knowledge would facilitate the development of targeted public health strategies to effectively reduce the global burden of chronic kidney disease.

## CONCLUSIONS

This analysis provides an up-to-date picture supporting secular improvements in global chronic kidney disease due to diabetes mellitus type 2 (CKD-T2DM) burden attributable to low physical activity (PA) and deterioration in that of high body-mass index (BMI) throughout the past 32 years. The extent of CKD-T2DM burden attributable to low PA and high BMI differs substantially by age, sex, and regions and especially shows strong association with socioeconomic status. The escalating burden of high-BMI-related CKD-T2DM in several regions, including Andean Latin America, Central Latin America, the Caribbean, Oceania, North Africa and the Middle East, as well as High-income North America, warrants heightened attention. It further highlights growth in early-onset CKD-T2DM burden attributable to low PA and high BMI. Targeted strategy from multidimensional efforts involving systemic interventions like community-based physically-active lifestyle promoting encouragement and appropriate policy interventions on weight management fitted to regional, temporal, and developmental background, is vital.

## Supplementary Material

Global burden of chronic kidney disease due to diabetes mellitus type 2 attributable to low physical activity and high body mass index from 1990 to 2021
