# Supplementary material for: Global burden of chronic kidney disease due to diabetes mellitus type 2 attributable to low physical activity and high body mass index from 1990 to 2021
Source: Biol Sport. 2025 May 8;42(4):171–86. doi: 10.5114/biolsport.2025.150045 (PMC12492348; doi:10.5114/biolsport.2025.150045)
Supplement: Global burden of chronic kidney disease due to diabetes mellitus type 2 attributable to low physical activity and high body mass index from 1990 to 2021 [file JBS-42-4-56024-s1.pdf]

Supplementary Figures

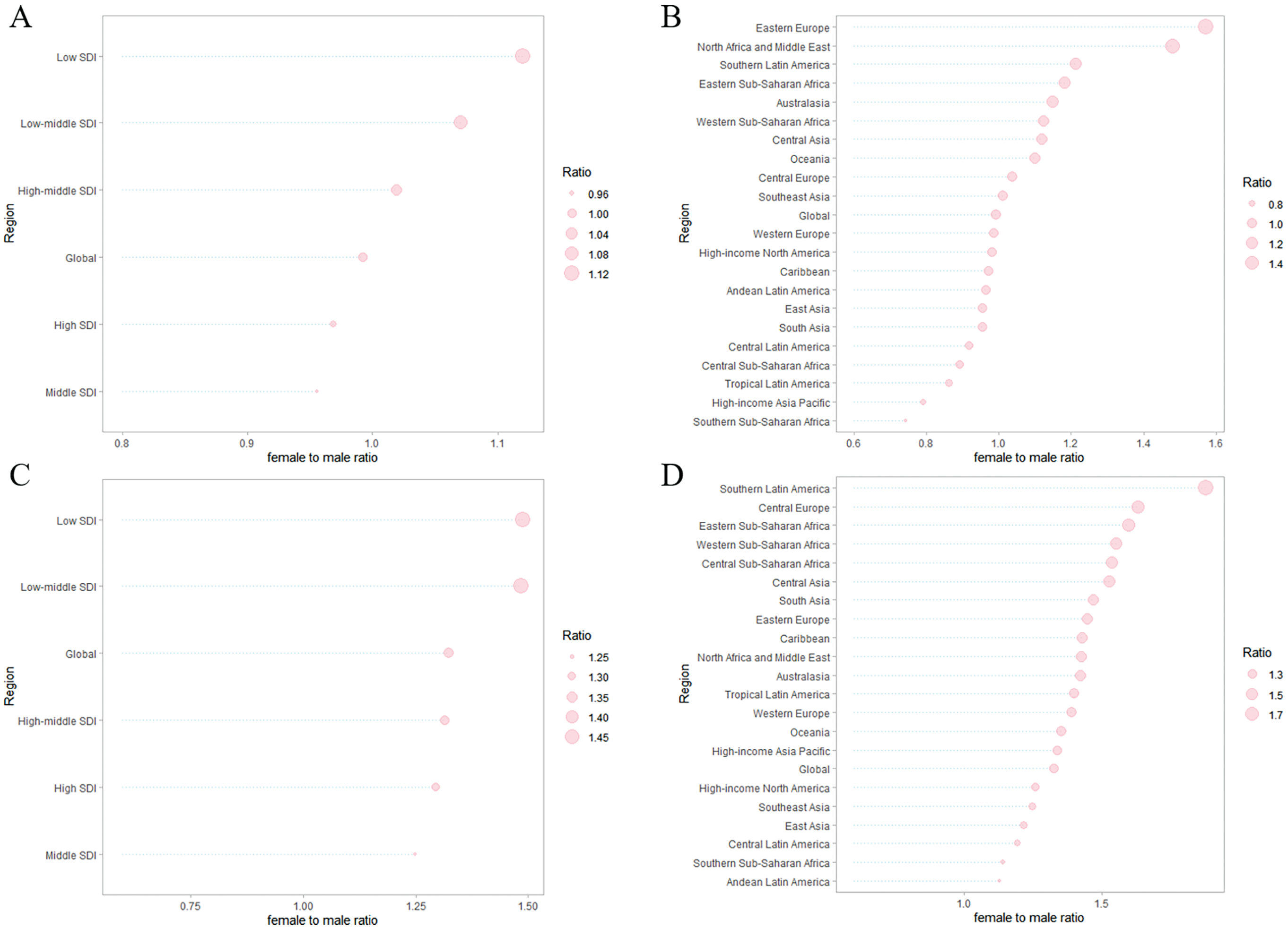

FIG. S1. Female to male ratio of low-PA-related age-standardized DALYs rate for CKD-T2DM across SDI regions in A, and that across 21 GBD regions in B, and corresponding female to male ratio of population attributable fractions in C and D, respectively

Articles published in the Biology of Sport are licensed under an open access Creative Commons CC BY 4.0 license.

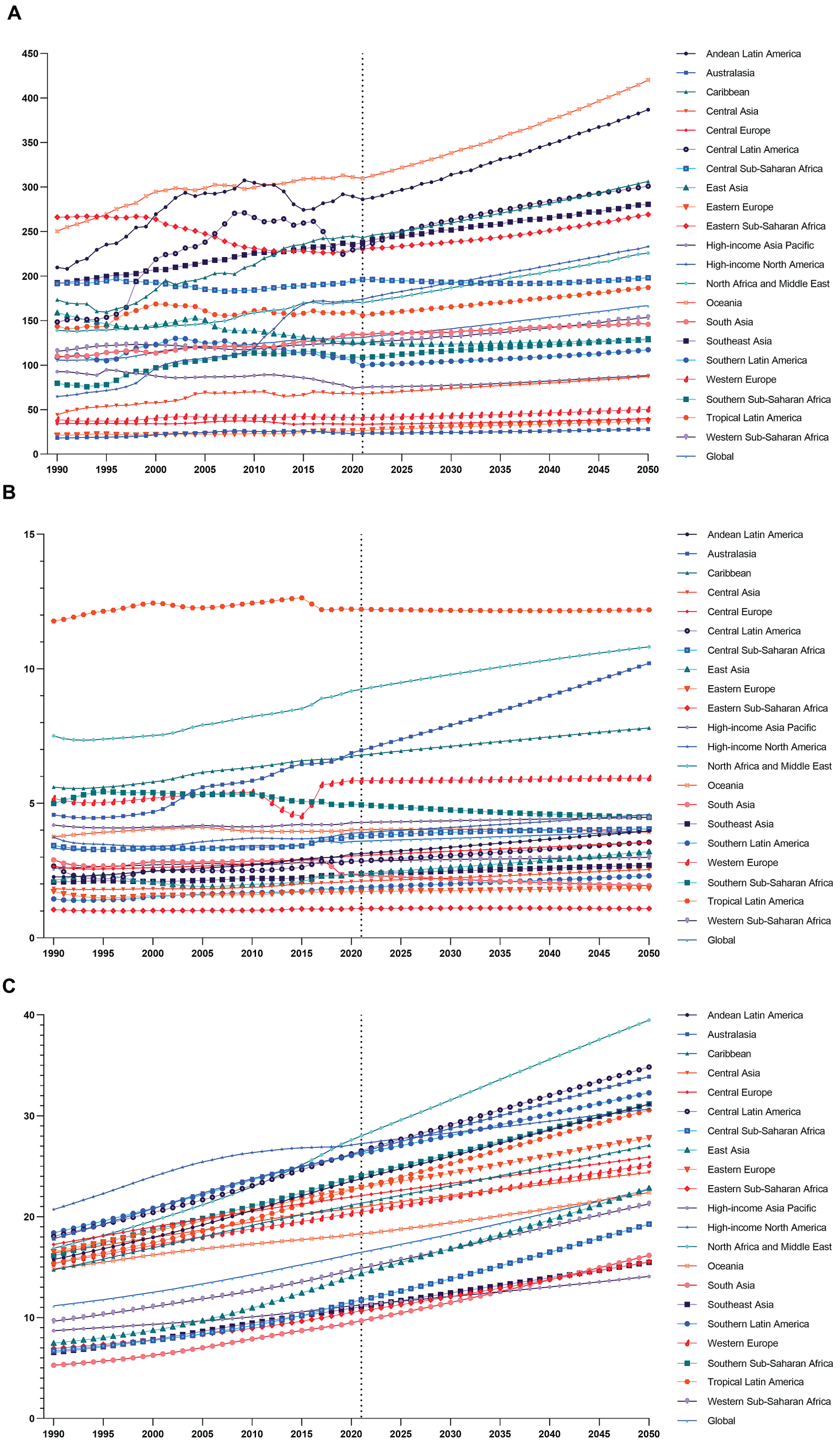

FIG. S2. Female to male ratio of high-BMI-related age-standardized DALYs rate for CKD-T2DM across SDI regions in A, and that across 21 GBD regions in B, and corresponding female to male ratio of population attributable fractions in C and D, respectively

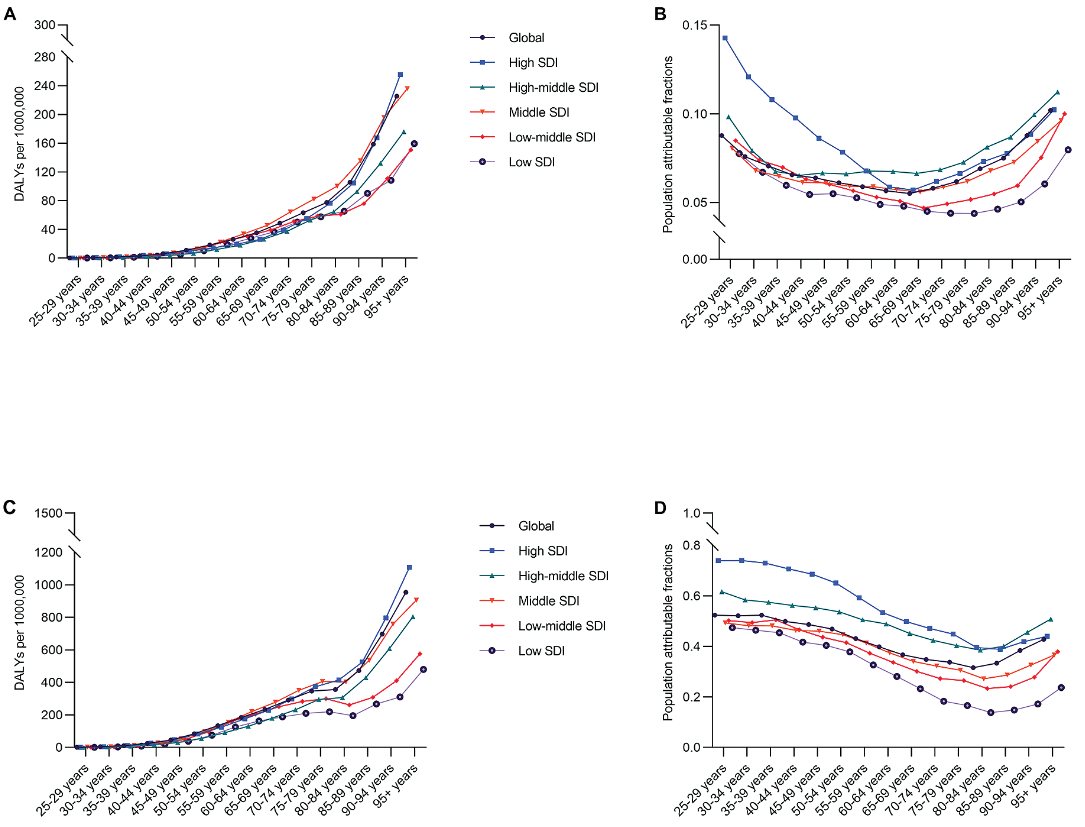

FIG. S3. Variation of DALYs rate for low-PA-related CKD-T2DM for each age group from GBD 2021 in A, and proportion of age-standardized DALYs for CKD-T2DM attributable to low PA on total CKD-T2DM for each age group in B, and corresponding age patterns of high-BMI-related CKD-T2DM burden for each age group in C and D, respectively

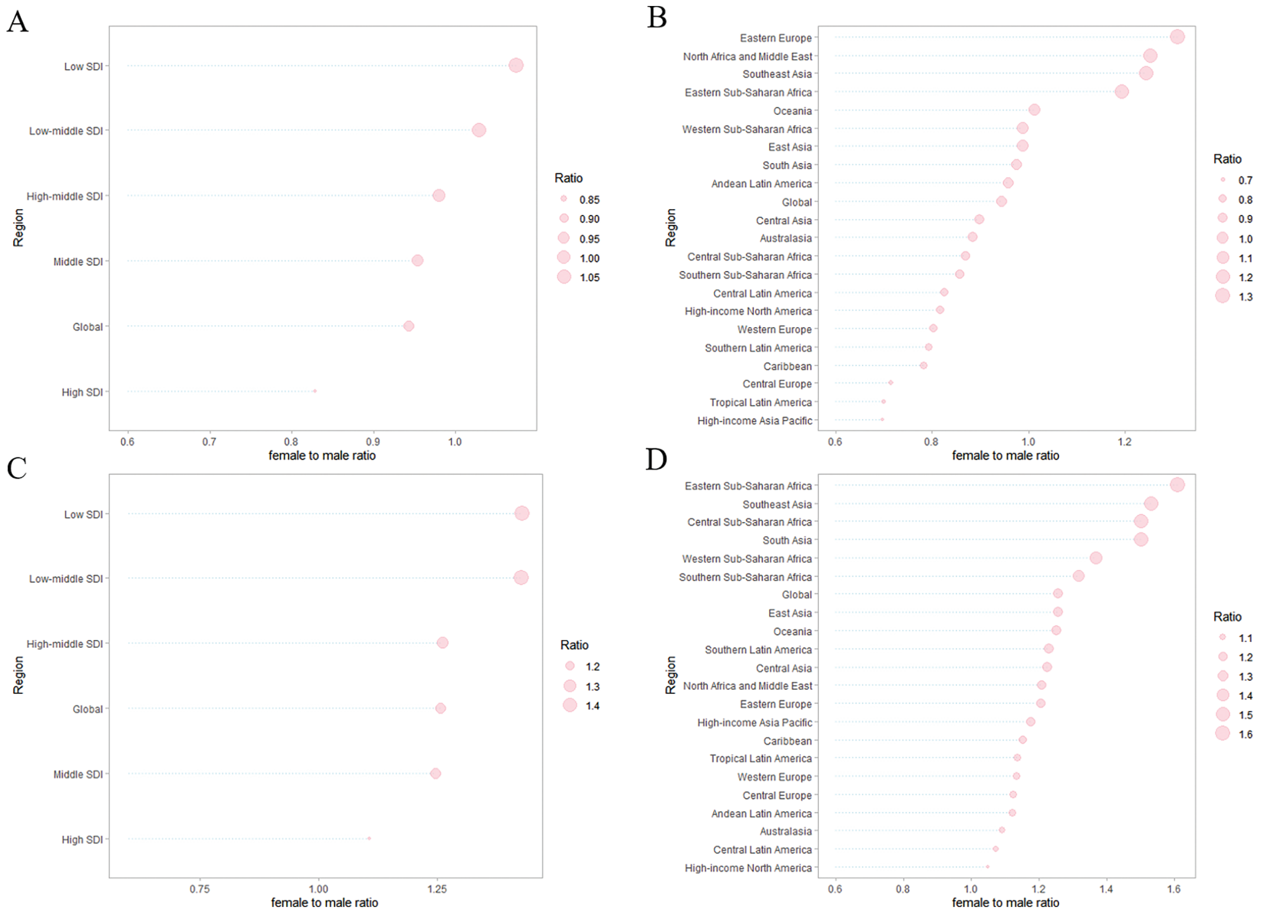

FIG. S4. Future forecasts of age-standardized rate DALYs for CKD-T2DM across 21 GBD regions untill 2050 in A, and prediction of low PA and high BMI exposure in B and C, respectively
